# Necrotizing pneumonia due to *Saprochaete capitata* in a patient with diabetes mellitus. Case report

**DOI:** 10.1016/j.mmcr.2023.08.006

**Published:** 2023-08-29

**Authors:** Alejandro Hernández Solis, Saul Javier Rabadan Armenta, Javier Araiza Santibáñez, Alexandro Bonifaz, Fryda Jareth Serna Valle, Eliasib Mojica Jaimes

**Affiliations:** a Servicio de Neumología y Cirugía de Tórax, Hospital General de México, “Dr. Eduardo Liceaga”, Ciudad de México, Código postal: 06720, Mexico; b Laboratorio de Micología, Hospital General de México, “Dr. Eduardo Liceaga”, Ciudad de México, Código postal: 06720, Mexico; c Universidad Anáhuac México, Ciudad de México, Código postal: 52786, Mexico; d Facultad de Estudios Superiores Iztacala, Universidad Nacional Autónoma de México, Estado de México. Código postal: 54075, Mexico

**Keywords:** *Saprochaete capitata*, Necrotizing pneumonia, Diabetes mellitus

## Abstract

*Saprochaete capitata* is a yeast-like fungus of the Dipodascaceae family, capable of colonizing the skin and the respiratory and gastrointestinal tracts. We present a 56-year-old man with diabetes mellitus who was admitted to the hospital presenting with fever, cough and hemoptysis. The diagnosis of necrotizing pneumonia was made by direct microscopy of the bronchoalveolar lavage fluid showed and *Saprochaete capitata* was identified by Matrix-Assisted Laser Desorption/Ionization Time-Of-Flight (MALDI-TOF MS®). Treatment consisted of itraconazole 200 mg every 12 hours orally for 30 days, leading to clinical and radiological improvement. *Saprochaete capitata* infection is a rare cause of pulmonary mycoses.

## Funding

This research did not receive any specific grants from funding agencies in the public, commercial, or nonprofit sectors.

## Conflict of interest

The authors declare that they have no conflict of interest.

## 1 Introduction

*Saprochaete capitata* (formerly known as *Geotrichum capitatum*) is a yeast-like fungus of the Dipodascaceae family, Saccharomycetales order. It is considered a highly virulent species located in the environment, mainly in soil, air, and water, and capable of colonizing the skin, the respiratory and gastrointestinal tract of humans, it can cause a rare infection known as geotrichosis, in patients with severe immunocompromise, the infection affects deep organs and can cause metastatic skin lesions, osteomyelitis, and hepatosplenic and brain abscesses.

Occasionally it can present in a context other than hematologic, causing prosthetic valve endocarditis, pneumonia, and meningitis associated with an overall mortality of 52–57% [[1], [2], [3], [4], [5], [6], [7], [8]].

According to the literature, 87% of the cases of infection by *Saprochaete capitata* have been reported in European countries on the Mediterranean coast, so it can be inferred that climatic conditions favor the development of this pathogen [[9], [10], [11], [12]]. Because of its clinical and radiological manifestations, *Saprochaete capitata* disease is often confused with *Mycobacterium tuberculosis* and *Candida* spp. infections, a reason why the number of cases reported might be underestimated [[3], [4], [5], [6], [7], [8],^13^].

Known as part of the normal microbiota, *Saprochaete capitata* does not represent a threat for immunocompetent individuals; nevertheless, immunocompromised patients, especially those having hematological malignancies, are at high risk of systemic spread. Currently there is no standardized treatment and due to antifungal drug resistance, it can be responsible for longer hospital stay and worsening prognosis [[2], [3], [4], [5]].

## 2 Case presentation

A 56-year-old male construction worker with 5-year diagnosis of type II diabetes mellitus, and poor adherence to insulin-based treatment, previous hospitalizations due to diabetic ketoacidosis and pneumonia due to SARS-CoV-2, 6 months earlier, with a hospital stay of 7 days, the oxygen saturation in ambient air showed fluctuations of less than 90%, for this reason supplemental oxygen was administered at 3 L per minute through non-rebreather reservoir bag oxygen mask, however he was discharged due to clinical improvement.

**Fig. 1:**
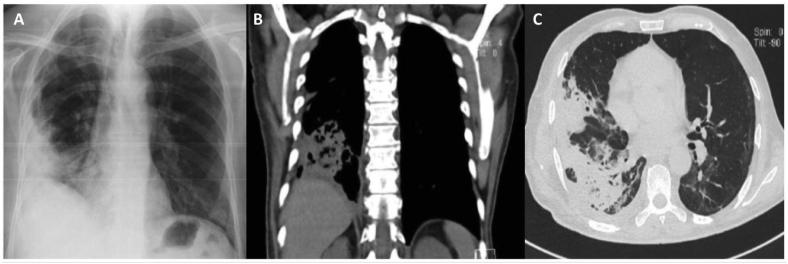
A) Posteroanterior chest X-ray: right basal heterogeneous opacity. B) Chest CT: hyperdense image, compatible with air consolidation. C) Chest CT: pulmonary opacity compatible with necrotizing pneumonia, in close contact with the pleura, with central cavitation.

**Fig. 2:**
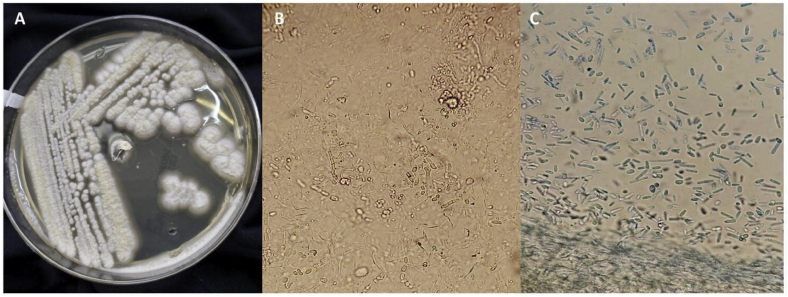
A) White, membranous, limited, rough and filamentous colonies, on Sabouraud's dextrose agar, development at 7 days, incubated at 30 °C. B) Hyaline septate filaments, arthroconidia and blastoconidia, on direct examination with 10% KOH (40x). C) Arthroconidia and blastoconidia of *Saprochaete capitata*, stained with lactophenol cotton blue (40x). (For interpretation of the references to colour in this figure legend, the reader is referred to the Web version of this article.)

After this the patient continued with recurrent episodes of cough and dyspnea, initially non-productive, the cough later was accompanied by a stinky, purulent sputum of about 150 ml per day (Day 0). The patient was admitted to the pulmonary care unit of a public teaching hospital in Mexico City (Day +10) with fever of 38.5 °C, hemoptysis of approximately 200 ml in 24 hours, a decrease in SpO2 to 88%, an elevated serum glucose (>300 mg/dl), and a Hb1c of 12%. Therefore, empirical treatment was started with a third-generation cephalosporin, but without effect on Day +12. Imaging showed right basal lung opacities with air consolidation corresponding to necrotizing pneumonia (Fig. 1). Results of cultures from bronchoalveolar lavage fluid (Day +20), Gene Xpert® and Ziehl-Neelsen stain were negative (Day +21). Sabouraud dextrose agar culture showed fungal growth, and direct examination with 10% KOH and lactophenol cotton blue stain showed hyaline septate filaments, arthroconidia and blastoconidia corresponding to *Saprochaete capitata* (Fig. 2) (Day +25). *S. capitata* was confirmed by Matrix-Assisted Laser Desorption/Ionization Time-Of-Flight (MALDI-TOF MS®, Bruker Daltonics Inc. Billerica, MA, USA). Susceptibility testing using the semi-quantitative Fungi Test® Bio-rad method, showed susceptibility to amphotericin B and itraconazole. The antibiotics were discontinued and itraconazole treatment was started 200 mg orally every 12 hours (Day +26), showing clinical improvement in clinical symptoms and chest imaging after 7 days of administration (Day +33). The patient was discharged home once oxygen support was no longer needed with SpO2 remaining above 92% in ambient air, no fever, and no sputum production, with treatment up to 30 days (Day +34). Follow-up at the outpatient clinic 3 and 6 months after hospital discharge, showed no recurrence.

## 3 Discussion

The route of entry of *Saprochaete capitata* can be endogenous as it forms part of the gut and oral microbiota, as well as exogenous if it is acquired from the environment by aspiration of arthroconidia, anneloconidia and blastoconidia. [14]

Spread via inhalation of contaminated air is feasible, as *S. capitata* belongs to a group of rare but emerging yeasts of significant importance. These yeasts can trigger both isolated cases and hospital outbreaks of Invasive Fungal Infections (IFI). Outbreaks in hospital settings, especially in patients with oncohematologic diseases, of IFI caused by *Saprochaete capitata* have been documented. These outbreaks have been related to the contamination of milk or its derivatives in thermal distribution containers. The various environmental media involved in the spread of rare spores include: malfunction of ventilation systems, poor maintenance of air filters, contamination of insulation materials, construction work in and around the hospital, leaks of water and humidity within the hospital infrastructure, contaminated food. [13]

The highest airborne spore counts were observed in patient bathrooms, suggesting that the spores may have been dispersed as aerosols from the shower devices. However, the clinical implications of these findings, which are based on the investigations of a specific group of investigators, have not yet been fully defined. [13]

Signs and symptoms of *Saprochaete capitata* infection could be unspecific and often confused with other entities such as tuberculosis and candidiasis, a reason why it could remain underdiagnosed. Only a few cases are documented, all of them involving the skin and associated with severe stages of immunosuppression such as in acute leukemia related neutropenia [9,12].

Currently around 250 cases of infection by this etiological agent have been reported, more than 90% in patients with leukemia and hematological diseases and in a lower percentage in patients with a non-hematological condition such as diabetes mellitus, connective tissue diseases, chronic kidney disease, and history of tuberculosis, all these pathologies are related to a deterioration of the immune system, however cases have also been reported in immunocompetent patients [1]. In a multicenter retrospective study, documented 35 cases of infections caused by *S. capitata*, localized variants were observed predominantly in the lungs (14.3%). Most of these infections occurred in patients with hematologic malignancies [15]. In the presented case, the patient had no cancer history but diabetes mellitus and previous history of SARS-CoV-2 pneumonia [3,10].

Not well treated diabetes mellitus patients present a chronic inflammatory state of constant recruitment and local activation of macrophages and neutrophils, and proinflammatory cytokine release. This inflammatory pattern could be enhanced by the immune response against viruses such as SARS-CoV-2, favoring fungal infections. Pre-existing comorbidities, the indiscriminate use of antibiotics, and inadequate control of secondary infections facilitate the appearance of systemic infections caused by fungi in predisposed individuals [16].

Lung infection presents a non-specific clinical picture, and there may be asthenia, adynamia, persistent and fever greater than 38°. Radiologically, the lesions show a pattern similar to the damage caused by agents such as *Mycobacterium Tuberculosis* and *Candida Spp*, which is why they are usually the first diagnoses. In the case of our patient, the results of the cultures of bronchoalveolar lavage fluid, Gene Xpert® and Ziehl-Neelsen staining were negative [2].

Mycological diagnosis is based on the detection of hyaline septate filaments, arthroconidia and blastoconidia on direct examination. The morphology of the *Saprochaete* species is identical, making it necessary to make use of advanced diagnostic modalities such as MALDI-TOF MS® or PCR sequencing of ITS and large partial subunit (LSU) loci that can discriminate between the two species, MALDI-TOF MS® was permorf in our patient to confirm the diagnosis [2].

Due to the scarcity of cases reported and poor experience in its pharmacological treatment, there is currently no standardized management for *Saprochaete capitata* infection. Echinocandin monotherapy is not recommended, as it is associated with increased mortality, showing broad resistance to these and low fluconazole susceptibility, on the other hand *Saprochaete capitata* has low Minimum Inhibitory Concentrations (MICs) for 5-flucytosine (MIC 0.125–0.5 mg/L), itraconazole (MIC range 0.03–0.5 mg/L), posaconazole (0.016–1 mg/L), voriconazole (0.03–0.5 mg/l). The MICs for amphotericin B tend to be in the moderate range (MIC 0.5–2 mg/L) therefore Amphotericin B alone or in combination with 5-flucytosine or voriconazole could be considered as the first choice of treatment [1]. Management of many rare fungal infections requires individualized approaches that take into account susceptibility results along with clinical presentation, in the case of our patient, the pathogen showed susceptibility to amphotericin B and itraconazole treatment, so treatment was given based on itraconazole showing a good clinical response [2,6,[9], [10], [11], [12]].

Both *Saprochaete capitata* pneumonia and pulmonary mycoses share similar radiological features, especially in immunosuppressed patients, as well as signs and symptoms characterized by productive cough, chest pain, dyspnea, and hemoptysis. Also, in the chronic forms of both diseases, asthenia, adynamia and weight loss occur, so it is common for an erroneous diagnosis to be made between both entities that could result in treatment delays, longer hospital stays, increased healthcare costs and worse prognosis. The reported mortality is from 52% to 57% [2].

Currently, the actual prevalence and incidence of pulmonary mycoses are unknown, particularly in limited resources countries due to the lack of effective diagnostic tests, leading to underdiagnosis and delayed diagnoses at advanced stages [4].

Understanding the etiology of endemic pulmonary mycoses is of paramount importance. Although histoplasmosis, coccidioidomycosis, paracoccidioidomycosis, blastomycosis, and sporotrichosis represent the most frequent, mucormycosis, aspergillosis, candidiasis and cryptococcosis affect the most those patients with diabetes mellitus, HIV/AIDS, cancer and chronic use of corticosteroids [17].

Not an infrequent but a barely reported disease, *Saprochaete capitata* infection represents an emerging health problem for immunocompromised patients. In our hospital environment, the reported cases are scarce, since most of the reports correspond to European countries on the Mediterranean coast [[9], [10], [11], [12],^15^]. It is important to implement diagnostic tools for the identification and timely management of fungal infections in clinical practice. [8]
